# Financial impact of pandemics on pension sustainability: an application for Spain

**DOI:** 10.1007/s10203-024-00482-w

**Published:** 2024-10-14

**Authors:** M. Carmen Boado-Penas, Julia Eisenberg, Zuochen Song

**Affiliations:** 1 Heriot-Watt University, Edinburgh, UK; 2 TU Wien, Vienna, Austria; 3 University of Liverpool, Liverpool, UK

**Keywords:** Pensions, Sustainability, Pandemics, COVID-19, Unemployment, Mortality, Fertility, H55, J11, J26

## Abstract

Epidemiologists are warning that the next pandemic is almost around the corner. As we have seen during the COVID-19 pandemic, the death toll was at the highest for over 60th, transforming the population pyramids. In times of pandemics, two effects on sustainability of the pay-as-you-go (PAYG) pension scheme go in different directions: the increase in old-age mortality (leading to a decrease in pension spending) and the increase in unemployment (leading to a decline in income from contributions). In this paper, we investigate the long-term effects of pandemics on the financial sustainability of PAYG pension schemes, taking into account changes in both mortality and unemployment factors. Using functional data analysis we develop projections of population pyramids and allow for anticipated mortality jumps as a result of future pandemics. An application is carried out using mortality and fertility data from Spain. Given some plausible assumptions, our findings indicate, firstly, that the financial sustainability of the Spanish pension system is compromised. Secondly, in the event of a pandemic, we observe that financial sustainability is primarily driven by the mortality effect—resulting in a decrease in pension expenditure—while the impact of unemployment is almost negligible.

## Introduction

Continuous improvements in medical and healthcare systems, along with falling birth rates, contribute to the increase in the old-age dependency ratio—usually defined as the ratio between the number of people aged 65 years and more over the number of working-age population (20–64 years). For the readers interested in health insurance we refer to Pitacco (2004).

According to Eurostat (2023), the old-age dependency ratio in the European Union is likely to double from 33.3$\%$ in 2023 to 59.7$\%$ by 2100. The proportion of people over 80 in countries such as Italy and Spain will increase from 7$\%$ to 20$\%$ by the end of the century, see the projections in United Nations (2022). In fact, even in the elderly age groups of around 90 years, mortality rates are declining rapidly, see Rau et al. (2008).

An increasing old-age dependency ratio can bring different challenges to a country, but, above all, it damages the financial sustainability of state pension schemes—generally financed on pay-as-you-go (PAYG) basis, where the current contributors cover the pensions for current retirees. Hence, the pension scheme is steadily losing its financial equilibrium due to an increasing number of pensioners in comparison to the contributors. The common trend in the responses to the pension crisis is a wave of parametric adjustments that usually include changes in the contribution ceilings, increases in the retirement age or changes in the indexation of pensions, see OECD (2021).^1^

A catastrophic event like a world war or a pandemic usually leads to an increase in the number of deaths and even changes the projected trajectory of mortality rates for several years, see, for instance, Liu and Li (2015). In 2020, COVID-19—the fifth worldwide pandemic documented after the Spanish influenza in 1918 (World Health Organization 2021)—caused millions of deaths and dramatically changed people’s lives. During COVID-19, a respiratory virus heavily infected older cohorts and people with co-morbidities, with age being proved as the most representative indicator of death, see Bhargava et al. (2021). In particular, 81% of the people who died from COVID-19 in the USA in 2020 were over 65 while those aged 90 and over were almost three times more likely to die from COVID than those aged 65 to 70, see Betzaida and Kramarow (2022). According to Rossen et al. (2022), the excess deaths caused by the pandemic raised mortality rates for Europeans aged over 60 by approximately 1$\%$ during the years 2020 and 2021, leading to a decrease in the old-age dependency ratio.

On the other hand, COVID-19 has demonstrated that governmental measures like protracted lockdowns can increase the unemployment rate, see COVID Hardship Watch (2022), and, therefore, reduce the income from contributions of state pension schemes. In addition, during COVID-19, some countries have even suspended, or reduced, contributions made by employees and/or employers and/or extended the eligibility of unemployment benefits to cover not only full but also partial employment, see Boado-Penas et al. (2022).

Therefore, during a pandemic one expects two conflictive effects on the financial sustainability of PAYG schemes. While the increase in old-age mortality would cause a decrease in pension spending, a higher rate of unemployment would have a negative impact on income from contributions. The overall effect on financial sustainability is not clear and needs a more detailed investigation.

The consequences of COVID-19 on mortality have been extensively studied. Cairns et al. (2020) found that many of those who died from COVID-19 would have died soon even without the virus due to the presence of certain co-morbidities—leading to a minimal change in life expectancy. They predict that mortality rates will return to pre-pandemic levels but acknowledge potential indirect deaths due to new impairments from the virus or delayed medical care during the pandemic. Lee et al. (2020) used generalized additive models to analyse US mortality data from March 2020 to the end of 2021. They found a major significant direct impact of COVID-19 on mortality from causes like diabetes, heart disease, and cerebrovascular diseases, especially in those over 45. Indirect effects predominate in mortality from external causes (i.e. suicides, opioid overdoses, and accidents) and all-cause mortality among individuals under 44. Stricter intervention periods are associated with greater increases in mortality for this younger age group.

Ruzzenenti et al. (2021) described both direct and indirect impacts of COVID-19 on cardiovascular diseases, noting a 58% increase in out-of-hospital cardiac arrests in Lombardy during the first 40 days of the outbreak compared to the same period the previous year—for more details, see Baldi et al. (2021).^2^

In the present paper, we aim to analyse the impact of future pandemics (with similar features to those of COVID-19) on the financial sustainability of state pension schemes—normally financed on a PAYG basis. With the aim of projecting population pyramids, we use functional data analysis to forecast both mortality and fertility rates, see Hyndman and Ullah (2007). Compared to the Lee-Carter model, this methodology can include more complex terms in historical data with less observational noise and thus produce models with higher accuracy.

Accounting for catastrophic events resembling COVID-19, we include mortality jumps into the projected mortality rates, relying on plausible assumptions about the jump frequency and size of these jumps. Using Spain as an illustration, we find that, firstly, the Spanish pension system is not financially sustainable, and secondly, its sustainability is primarily driven by the mortality effect that generates a decrease in the pension expenditure, while the increase in unemployment resulting from the pandemic has a negligible impact.

The paper is structured as follows. In Sect. 2, we describe the functional data analysis to project fertility and mortality rates together with the methodology to construct population projections. In Sect. 3, we show results of population projections and pension sustainability for the case of Spain. In Sect. 4, a sensitivity analysis is carried out. Specifically, we analyse the results under different choices of fertility rates, mortality jump sizes and frequencies, and unemployment levels. We conclude in Sect. 5 and provide insights into potential directions for future research.

## Methodology and data

In this section, we present the mortality and fertility data for Spain used in this study. Additionally, we detail the methodology, that includes

- Fitting models and producing forecasting for mortality and fertility rates;
- Introducing mortality jumps to the model;
- Producing projected population pyramids.

### Remark 1

Patterns of migration are rapidly changing due to economic, political, as well as climate-related factors, making migration a very difficult variable to analyse and forecast. In the specific case analysed in Sect. 3, the net migration values for Spain are projected to be relatively low for most year from 2022 to 2071—see INE (2023). Therefore, we do not use this variable when calculating the projected population pyramids.

### Data sources

Mortality and fertility data spanning the period from 1970 to 2020 for both females and males in Spain are used. Mortality data is collected from Human Mortality Database (2023b), world’s leading scientific data resource on mortality in developed countries, providing comprehensive information on mortality and population dynamics. The data is divided into 5-year age groups, ranging from age 0 to 110+, denoted as, 0, 1–4, 5–9, $\ldots$, 105–109 and 110+. In this paper, these groups are identified as age groups 1 to 24.

On the other hand, fertility data is obtained from Eurostat (2023c) and covers individuals aged 16 to 50 years old. The fertility data is categorized into groups: 16–19, 20–25, $\ldots$, 40–45 and 46–50—with these groups numbered from 5 to 11.

**Fig. 1:**
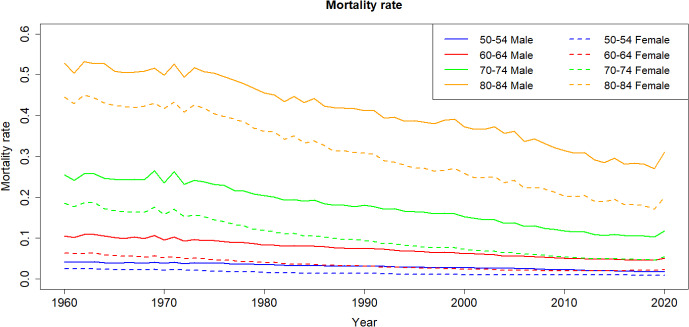
Mortality rates from 1960 to 2020 in Spain

**Fig. 2:**
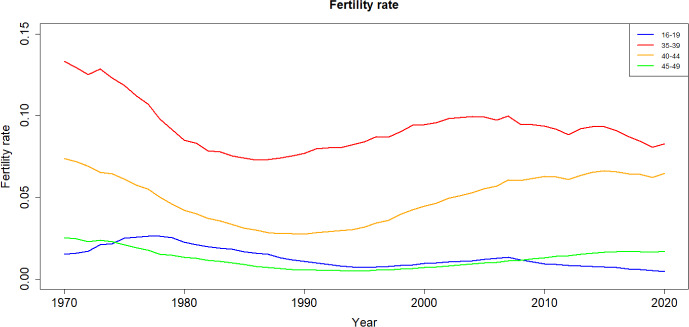
Fertility rates from 1960 to 2020 in Spain

Figs. 1 and 2 illustrate the historical trends in mortality and fertility rates over time. It can be seen, Fig. 1, that the mortality rates have exhibited a consistent decline across all age groups in recent decades. Furthermore, we observe that men tend to have a higher average mortality than women within the same age group.

In Fig. 2, the trajectory of fertility rates after the year 1980 displays significant variation among different age groups. Therefore, it would be inaccurate to claim a consistent decrease in fertility in recent year. For instance, the fertility rate among females aged 40–44 has actually increased since 1990. However, it is important to acknowledge the exiting disparities in fertility rates among various age groups.

Additionally, for a more comprehensive analysis of the financial sustainability of the pension scheme, we include unemployment rates from the INE (2023) for both females and males. The data presented in Table 1 highlights the unemployment rates for both genders in the years 2019 and 2020. We can see that, overall, the unemployment rates have increased from 2019 to 2020, which coincides with start of the pandemic in early 2020.

**Table 1:** Unemployment rates ($\%$) of different age groups in the years 2019 and 2020 in Spain

Age group	2019 Female	2020 Female	2019 Male	2020 Male
16–19	48.47	60.97	42.82	50.11
20–24	31.65	36.22	28.18	34.23
25–29	19.10	23.69	18.87	22.16
30–34	16.34	18.67	11.66	15.08
35–39	13.46	16.68	9.68	11.79
40–44	13.97	14.61	9.22	9.87
45–49	13.92	14.79	9.48	10.24
50–54	14.85	14.22	10.44	10.63
55–59	13.82	14.26	11.68	11.01

For unemployment rates in the future, we assume that after the mortality jump, the unemployment rate is assumed to decrease following the pattern observed during COVID-19, and would use official projections afterwards.

### Mortality and fertility models

For modelling mortality and fertility rates, we follow the method introduced in Hyndman and Ullah (2007), since it allows more complex dynamics and reduces the observational noise with non-parametric smoothing. The model uses basis functions to estimate coefficients and to forecast mortality and fertility rates. This method can be summarized as follows:

1. Let $y_t(x)$ denote the log of the mortality or fertility rate for age *x* at time *t* with an underlying smooth function $f_t(x)$,
  1 $$\begin{aligned} y_t(x)=f_t(x)+\sigma _t(x)\epsilon _{t,x}, \end{aligned}$$
  where $\epsilon _{t,x}$ is sequence of i.i.d. standard normally distributed random variables.
2. The data is smoothed for each *t* using a nonparametric smoothing method to estimate $f_t(x)$.
3. The fitted curves are decomposed with a basis function expansion with the following model
  2 $$\begin{aligned} f_t(x)=\mu (x)+\sum ^K_{k=1}\beta _{t,k}\phi _k(x)+e_t(x), \end{aligned}$$
  where $\mu (x)$ is a measure of location of $f_t(x)$, $\phi _k(x)$ is a series of orthonormal basis functions and $e_t(x)\sim N(0,v(x))$.
4. Fit the time series models to each of the coefficients $\beta _{t,k}$ and forecast them using the fitted time series models.
5. Use the estimated coefficients with functions in (1) and in (2) to get forecast for $f_t(x)$ and $y_t(x)$.

Note that this modelling framework can be applied using the “demography” package in R.

### Introducing mortality jumps

As suggested by Chen and Cox (2009), when considering a mortality jump as a transitory event, the probability of a mortality jump in a given year is estimated to be 0.0436 for the U.S. population. We adopt this probability of a mortality jump for our numerical illustration. Specifically, we assume that a mortality jump would occur every 20 years and have a significant effect on mortality for two following years after each jump—similarly to COVID-19 pandemic. In other words, in our baseline scenario, we assume that any forthcoming pandemic would result in a similar increase in number of deaths per age group as witnessed during the COVID-19 pandemic.

We acknowledge that mortality rates can vary and depend on various factors, as described in Pitacco (2019). However, our analysis focuses on cases that can be compared to COVID-19.

Considering the number of deaths attributed to COVID-19 in 2020, Eurostat (2023a, 2023b), we compute the adjusted number of deaths during the first and second year following future pandemics as follows:

$$\begin{aligned} D_{t_1}^{*(g)}(j)=D_{t_1}^{(g)}(j)\cdot \frac{D_{2020}^{(g)}(j)}{\bar{D}_{2019}^{(g)}(j)},\\ D_{t_2}^{*(g)}(j)=D_{t_2}^{(g)}(j)\cdot \frac{D_{2021}^{(g)}(j)}{\bar{D}_{2019}^{(g)}(j)}, \end{aligned}$$

where $\bar{D}_{2019}^{(g)}(j)$ is the average "smoothed" death number in age group *j* between year 2016 to 2019 for gender *g*, $D_{t_1}^{(g)}(j)$, $D_{t_2}^{(g)}(j)$ are the projected death numbers of age group *j* ($j=1,2,\ldots , 24$) in the first and second year after the pandemic, respectively, based on pre-pandemic calibration and $D_{t_1}^{*(g)}(j)$, $D_{t_2}^{*(g)}(j)$ are the death numbers considering mortality jumps.

### Construction of population projections

In order to generate population projections, we employ a matrix notation using a Leslie matrix, see Leslie (1945).

A Leslie matrix is a square matrix used for population projections and growth analysis that consider survival and fertility rates. The general form of the matrix can be defined as

$${\textbf{P}}[t]$$=$$\begin{bmatrix} f_{0,t}& \quad f_{1,t}& \quad f_{2,t}& \quad \cdots & \quad f_{n-1,t}& \quad f_{n,t} \\ s_{0,t}& \quad 0& \quad 0& \quad \cdots & \quad 0& \quad 0 \\ 0& \quad s_{1,t}& \quad 0& \quad \cdots & \quad 0& \quad 0 \\ \vdots & \quad \vdots & \quad \vdots & \quad \ddots & \quad \vdots & \quad \vdots \\ 0& \quad 0& \quad 0& \quad \cdots & \quad s_{n-1,t}& \quad 0 \end{bmatrix},$$

where $f_{i,t}$ represents the fertility rate for females at ages *i* and time *t* ($f_i\ge 0$,$i=0,1,2,\ldots ,n$) and $s_{i,t}$ is the annual survival probability at age *i* and time *t* ($1\ge s_i\ge 0$).

Let ${\textbf{N}}[t]$ be a column vector with entries given by the number of individuals alive at age *i* at time *t*, i.e.

$$\begin{aligned} {\textbf{N}}[t]=(l_{0,t},l_{1,t},l_{2,t},\ldots ,l_{n,t})^T. \end{aligned}$$

Then, it holds that

$$\begin{aligned} {\textbf{P}}[t]{\textbf{N}}[t]={\textbf{N}}[t+1]. \end{aligned}$$

The first row of the matrix above will generate the number of newborns in the population and the rest of the rows will indicate the number of survivors through the year.

In our study, since the mortality and fertility data are in 5-year age groups, the Leslie matrix between year *t* and $t+5$ is updated for each gender separately as follows,

$${\textbf{P}}^{(g)}$$[t]=$$\begin{bmatrix} 0& \quad 0& \quad 0& \quad k^{(g)}F_{t+5}(5)\frac{L_{t+5}^{(f)}(5)}{L_{t}^{(f)}(4)}& \quad k^{(g)}F_{t+5}(6)\frac{L_{t+5}^{(f)}(6)}{L_{t}^{(f)}(5)}& \quad \cdots & \quad 0& \quad 0 \\ \frac{L_{t+5}^{(g)}(2)}{L_{t}^{(g)}(1)}& \quad 0& \quad 0& \quad 0& \quad 0& \quad \cdots & \quad 0& \quad 0 \\ 0& \quad \frac{L_{t+5}^{(g)}(3)}{L_{t}^{(g)}(2)}& \quad 0& \quad 0& \quad 0& \quad \cdots & \quad 0& \quad 0 \\ \vdots & \quad \vdots & \quad \vdots & \quad \vdots & \quad \vdots & \quad \ddots & \quad \vdots & \quad \vdots \\ 0& \quad 0& \quad 0& \quad 0& \quad 0& \quad \cdots & \quad \frac{L_{t+5}^{(g)}(24)}{L_{t}^{(g)}(23)}& \quad 0 \end{bmatrix}$$,

where

- $L^{(g)}_t(j)$ is the group population for gender *g* and group *j* at time *t*, *j*= 1, 2, $\cdots$, 24, *g*= *f* (for female), *m* (for male);
- $F_{t}(j)$ is the fertility rate at time *t* for age group *j*, *j*= 5, 6, $\cdots$, 11;
- $k^{(g)}$ is a constant to determine the number of newborns of both genders with
  $$\begin{aligned} k^{(f)}=\frac{SRB}{1+SRB}\quad \text{ and }\quad k^{(m)}=\frac{1}{1+SRB}, \end{aligned}$$
  where SRB is the sex ratio at birth and estimated at 1.07 using historical population from Human Mortality Database (2023b).^3^

For the purpose of constructing population projection, let ${\textbf{N}}^{(g)}[t]$ be a column vector of population for gender *g* by age group at time *t*, then we have

$$\begin{aligned} &  {\textbf{P}}^{(g)}[t]{\textbf{N}}^{(g)}[t]={\textbf{N}}^{(g)}[t+5], \\ &  {\textbf{N}}^{(g)}[t]=(L^{(g)}_t(1),L^{(g)}_t(2),L^{(g)}_t(3),\ldots ,L^{(g)}_t(24))^T \end{aligned}$$

To calculate the group population $L^{(g)}_t(j)$ from the matrix, we calculate the central death rate $m^{(g)}_t(j)$ for age group *j* and gender *g* at time *t* as follows

$$\begin{aligned} m^{(g)}_t(j) = -\ln (1-q^{(g)}_t(j)). \end{aligned}$$

According to Human Mortality Database (2023a) for the majority ages, mortality is approximately uniformly distributed within each of the 5-year age groups. Consequently, the group population size can be determined by averaging the population size of the first and last ages within that age group.^4^ Let $_t{\hat{L}}^{(g)}_x$ represent the population between age *x* and $x+1$ for gender *g* and at time *t*. For the first and last ages within each of our age groups, the 1-year population size can be computed as follows

$$\begin{aligned} &  _t{\hat{L}}^{(g)}_1= {_{t-1}{\hat{L}}}^{(g)}_0\cdot (1-m^{(g)}_{t-1}(1)), \\ &  _t{\hat{L}}^{(g)}_4={_{t-4}{\hat{L}}}^{(g)}_0\cdot (1-m^{(g)}_{t-4}(1))\cdot \prod ^3_{i=1}(1-m^{(g)}_{t-i}(2)), \\ &  _t{\hat{L}}^{(g)}_5={_{t-5}{\hat{L}}}^{(g)}_0\cdot (1-m^{(g)}_{t-5}(1))\cdot \prod ^4_{i=1}(1-m^{(g)}_{t-i}(2)), \\ &  _t{\hat{L}}^{(g)}_{5x}={_{t-5}{\hat{L}}^{(g)}_{5x-5}}\cdot \prod ^5_{i=1}(1-m^{(g)}_{t-i}(x+1)), x=2,3,\ldots ,24, \\ &  _t{\hat{L}}^{(g)}_{5x+4}={_{t-5}{\hat{L}}^{(g)}_{5x-1}}\cdot \prod ^5_{i=1}(1-m^{(g)}_{t-i}(x+2)), x=1,2,\ldots ,24. \end{aligned}$$

where $_t{\hat{L}}^{(g)}_0$ is the population between age 0 and 1 at time *t* and for gender *g* and can be calculated using the fertility rate and population size at time $t-1$.

After obtaining the 1-year population sizes, we can calculate the group population $L^{(g)}_t(j)$ as follows,

$$\begin{aligned} L^{(g)}_t(j)={\left\{ \begin{array}{ll} _t{\hat{L}}^{(g)}_0,j=1\\ \frac{4}{2}(_t{\hat{L}}^{(g)}_1+{_t{\hat{L}}^{(g)}_4}),j=2\\ \frac{5}{2}(_t{\hat{L}}^{(g)}_{5j-10}+{_t{\hat{L}}^{(g)}_{5j-6}}),j=3,4,\ldots ,23\\ _t{\hat{L}}^{(g)}_{110},j=24 \end{array}\right. }. \end{aligned}$$

For PAYG pension schemes, it is crucial to monitor the ratio between pensioners and contributors in the future to ensure the financial sustainability of the scheme. Therefore, we calculate the old-age dependency ratio *DR* as the ratio between the number of individuals aged 65 and older and those between the ages 20 and 64—using the projected population data, i.e:

$$\begin{aligned} DR_t=\frac{\sum ^{24}_{j=15}L_t(j)}{\sum ^{14}_{j=6}L_t(j)}. \end{aligned}$$

## Numerical results

Following Sect. 2.2, we provide results about projected mortality, fertility and population projections with and without mortality jumps.

### Mortality model

**Fig. 3:**
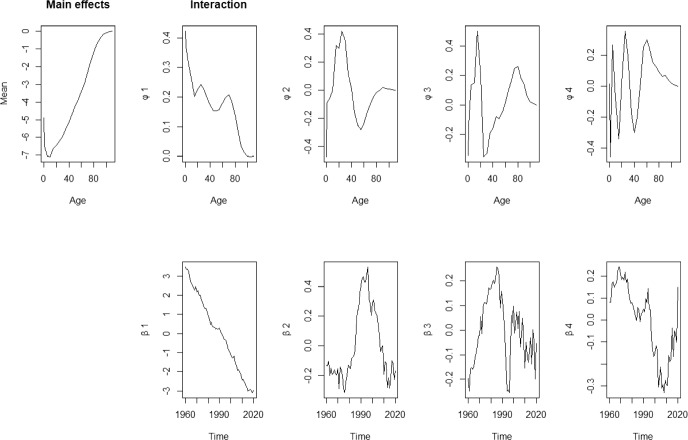
Basis functions and estimated coefficients for mortality rates for female population

**Fig. 4:**
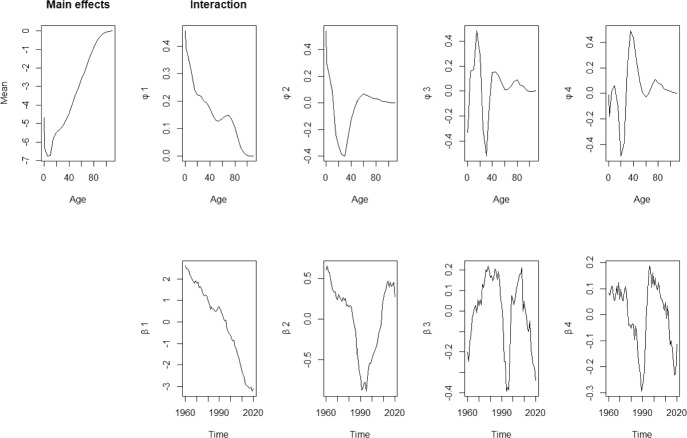
Basis functions and estimated coefficients for mortality rates for male population

For mortality rates, a decomposition of order $K = 4$ is used following Hyndman and Ullah (2007) in order to minimise the integrated squared forecast error. Figures 3 and 4 show the fitted basis functions $\hat{\phi }_k(x)$ and the corresponding coefficients $\hat{\beta }_{t,k}$ for female and male population in Spain, respectively.The basis functions model changes in mortality rates.

For the female population, the basis functions explain 96.76%, 1.59%, 0.52% and 0.33% of the variation, leaving only 0.8% unexplained. As seen in Fig. 3, these basis functions capture mortality rates across different age groups: ${\phi }_1(x)$ primarily represents individuals under age 80, with a focus on younger groups; ${\phi }_2(x)$ and ${\phi }_3(x)$ capture mortality changes in teenagers and those over 60. However, ${\phi }_4(x)$ is more complex, providing information across all age groups.

For the male populations, Fig. 4, the basis functions explain 91.58%, 6.46%, 0.95% and 0.42% of the variation, leaving only 0.59% unexplained. Figure 4 indicates that ${\phi }_1(x)$ and ${\phi }_2(x)$ capture mortality information for younger groups, while ${\phi }_3(x)$ and ${\phi }_4(x)$ focus on adults over 20. Since the first basis function, ${\phi }_1(x)$, contains most of the information for both genders, it is evident that overall mortality rates have been decreasing over the past 60 years, as indicated by the consistently decreasing of the coefficient ${\beta }_{t,1}$.

**Fig. 5:**
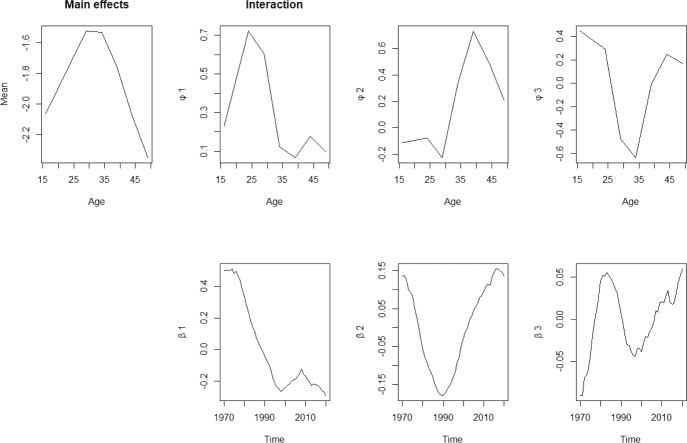
Basis functions and estimated coefficients for fertility rates (for women)

Following Hyndman and Ullah (2007), a decomposition of order $K=3$ is used for modelling the fertility rates. In Fig. 5, the basis functions explain 83.03%, 14.11% and 1.99% of the mortality variation, respectively, leaving only 0.87% unexplained.

These basis functions model fertility rates for females across different age ranges: ${\phi }_1(x)$ models rates for females under age 30, ${\phi }_2(x)$ models fertility rates for females around age 40, and ${\phi }_3(x)$ focuses on fertility rates of women in their 20 s and those over age 40. The coefficient associated with each basis function also illustrates changes in fertility rates before 2020. For instance, the coefficient ($\beta _{t,1}$) mainly decreases after 1970, while coefficient $\beta _{t,2}$ increases from 1990 onward, indicating a shift towards later childbearing ages. However, since the first basis function contains the majority of the model’s information, it is evident that the overall fertility rate is decreasing based on its coefficient.

**Fig. 6:**
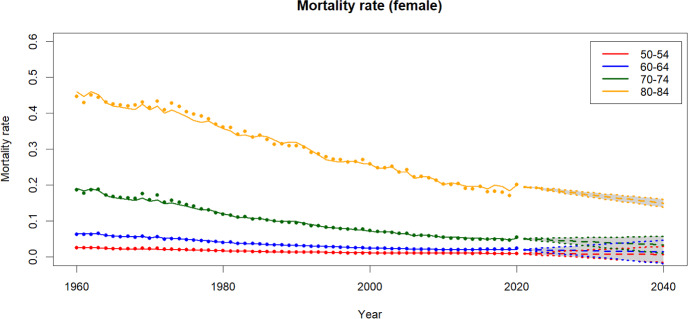
Historical, fitted and forecasts of female mortality rates

**Fig. 7:**
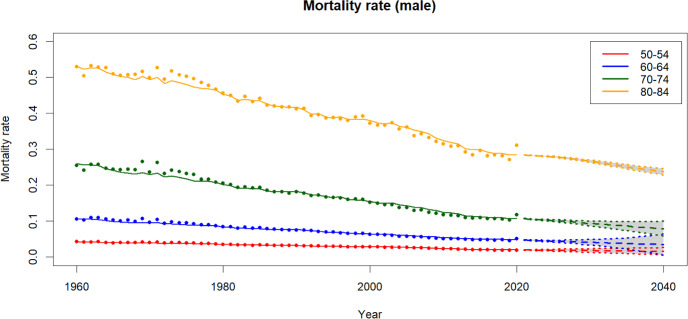
Historical, fitted and forecasts of male mortality rates

**Fig. 8:**
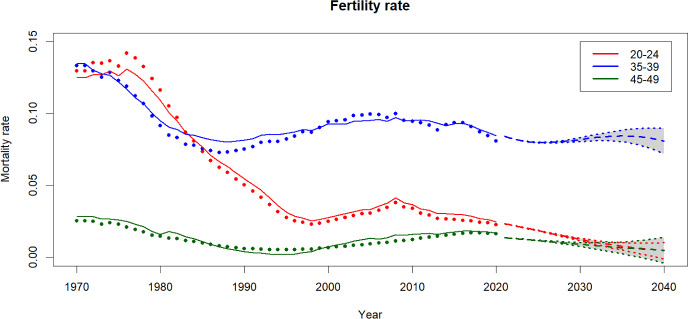
Historical, fitted and forecasts of fertility rates

**Fig. 9:**
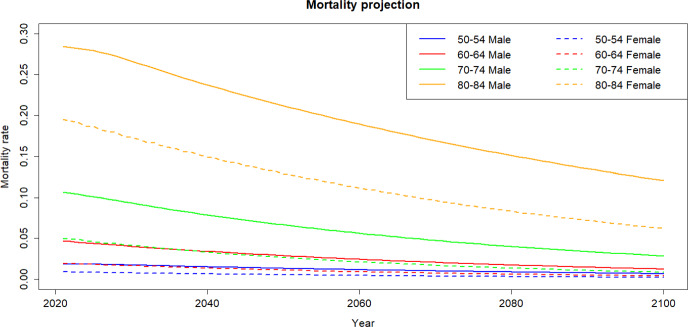
Projected mortality rates by age group

Figures 6 and 7 present mortality rates derived from historical data alongside the fitted values. The dots in the plots represent historical data, while the lines illustrate the mortality rates for different age groups during the data period using the fitted model. Overall, it can be observed that the models effectively capture the historical trend, with only minor discrepancies in the older age groups. Additionally, the projected mortality rates and their corresponding 90% confidence intervals are shown in the plots. On the other hand, Fig. 8 illustrates the historical, fitted values (solid line) and forecasts of the fertility rates.

### Mortality and fertility projections

Using the mortality and fertility models outlined in Sect. 2.2, projected rates for mortality for both genders and fertility rates are produced. In this paper, the forecast period spans 80 years.

In Fig. 9, we observe that women exhibit lower mortality rates compared to men within the same age group. The gender disparity in mortality rates escalates with age. For instance, for the 50–55 age group the mortality difference is around 0.01 but it can surge to 0.10 for individuals aged between 80 to 85 years. Projections indicate an anticipated decline in mortality rates for all age groups and both genders in the future.

**Fig. 10:**
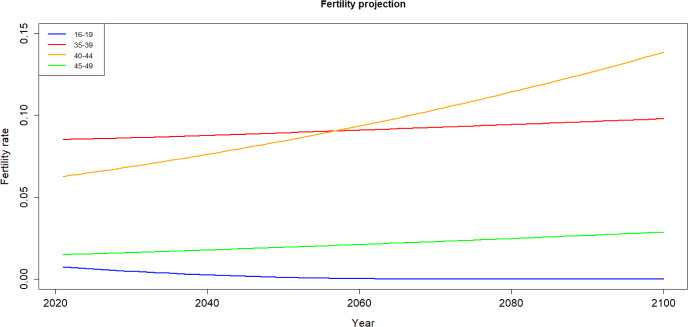
Projected fertility rates by age group

The fertility rates depicted in Fig. 10 hardly fluctuate within the same age group, with only a slight increase observed in older age groups. Particularly noteworthy is the notable rise in fertility rate—surge attributed to a consistent increase in the fertility rate for this group since 1990 as shown in Fig. 8.

### Population projections

**Fig. 11:**
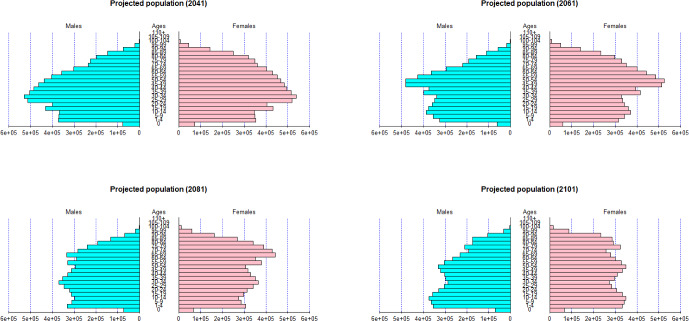
Projected population pyramids in 2041, 2061, 2081 and 2101

Once the forecasts of the mortality and fertility have been obtained, the next step involves generating projected population pyramids by gender. Figure 11 represents the projected population in 20, 40, 60 and 80 years respectively. Older age groups exhibit a higher proportion for both genders at the start of the forecast period. After several decades, the proportion of younger age groups increases in the population due to a slowdown in mortality rates among older individuals and a concurrently stable fertility rate. As a result, the old-age dependency ratio, as indicated in Table 2, increases up to 0.4003 in 2081 before gradually declining to a value of 0.3407 in 2101. Gender-specific differences are also observed, with women displaying a higher dependency ratio, attributed to their longer average lifespan.

Note that the values of the projected population pyramids may differ from other sources such as the Spanish National Institute of Statistics^5^ due to the use of different data sources. In addition, in our analysis, we do not consider migration.

**Table 2:** Old-age dependency ratio in 2041, 2061, 2081 and 2101

Gender	2041	2061	2081	2101
Total	0.2505	0.2750	0.4003	0.3407
Female	0.3018	0.3389	0.4937	0.4190
Male	0.1966	0.2073	0.3037	0.2615

### The effect of mortality jumps on pension sustainability

As mentioned in Sect. 2.3, a mortality jump is assumed to occur every 20 years during a time horizon of 80 years. That is, we assume that the jumps happen in years 2041, 2061 and 2081 and their consequences last for 2 years, with the size of the mortality jump similar to that of the COVID-19 pandemic per age-group and gender.

**Fig. 12:**
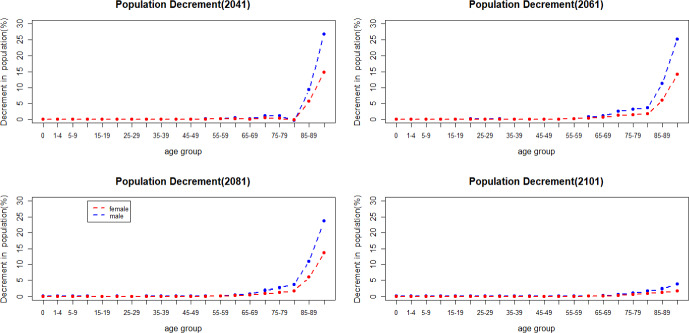
Percentage of population decrement caused by mortality jumps

Figure 12 shows the population decline when there is a jump in mortality in 2041, 2061, 2081 and 2101. Historical data from Eurostat (2023b) indicate that the increase in mortality caused by COVID-19 between 2020 and 2021 has had more impact on men in older age groups. As shown in Fig. 12, men have a more substantial increase in deaths in older age groups when a jump in mortality happens.

To calculate the financial equilibrium of the scheme certain assumptions must be made to calculate both the income from contributions and pension expenditure.

- We use the forecast demographic structures calculated in the previous sections.
- Current Spanish salary structure in used. Salaries are assumed to increase at an annual constant rate of 2%.
- For the calculation of pension expenditure we assume all individuals aged 65 and above receive the average pension.^6^
- Average pensions are assumed to increase at an annual constant rate of 2%. According to the Economic Bulletin (2022), public pensions in the Euro area are typically indexed automatically—either fully or partially—to prices and wages, mostly in a backward-looking manner, across almost all countries. Currently, public pensions in Spain are fully indexed to prices. The forecast for both inflation and GDP growth in Spain for 2025 is 2%, which is expected to translate into corresponding salary growth.^7^
- Projected unemployment rates for the case of Spain are used over the period 2022–2026, Statista (2023), and constant afterwards. In the event of a pandemic, unemployment rates observed during the COVID-19 pandemic over the period 2020–2021 are applied. Upon the conclusion of the pandemic, the unemployment level reverts to its normal state.
- We calculate the contribution rate to be paid by the individuals over the next 80 years as the one that equates income from contributions to pension expenditure in 2020. This computation yields a value of 25%.

**Fig. 13:**
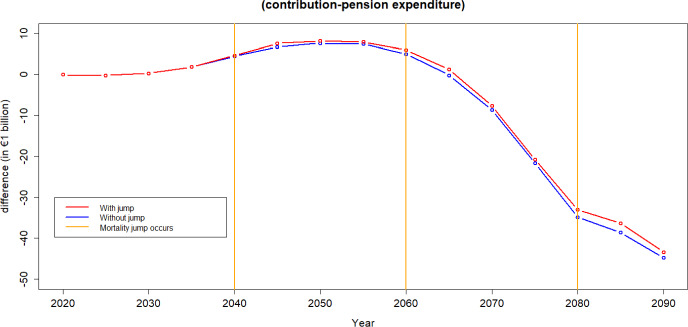
The difference between income from contributions and pension expenditure in Spain over time

Figure 13 shows the annual difference between the income from contributions and pension spending,^8^ i.e., the balance each year. The blue line does not include jumps resulting from a pandemic while the red line incorporates them. We build the population pyramids every 5 years and we can see how the equilibrium of the system is quite stable during the next two decades and worsens afterwards. Note that for this illustration the contribution rate chosen for the whole period is the one that makes the system balanced at the beginning of the period of analysis.^9^

**Fig. 14:**
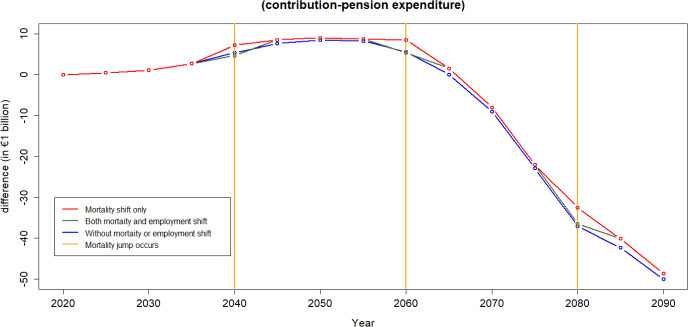
Difference between income from contributions and pension expenditure: Evaluating the mortality shift

When accounting for mortality jumps, a temporary enhancement in pension finances is observable, but this improvement quickly diminishes. It is essential to recognize that during pandemics, two conflicting effects impact sustainability: the increase in mortality, leading to a reduction in pension spending, and the increase in unemployment—which causes a decrease in income from contributions.

Figure 13, according to the assumptions applied in this analysis, shows that financial sustainability is mainly driven by the mortality effect that generates a decrease in the pension expenditure.

**Fig. 15:**
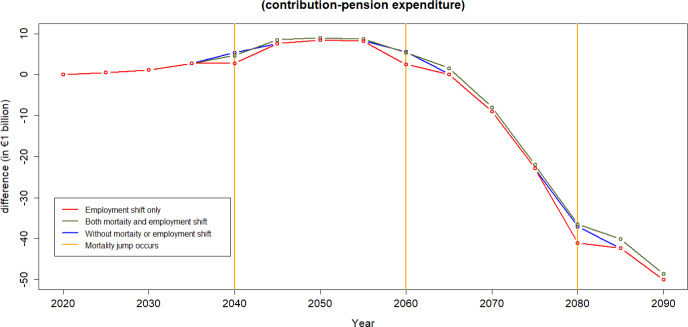
Difference between income from contributions and pension expenditure: Evaluating the employment shift

Figures 14 and 15 illustrate the contribution-expenditure differences considering either a shift in mortality or a shift in unemployment. If a pandemic causes only an increase in unemployment, the contribution base diminishes. Conversely, if a pandemic leads to only increased mortality of older age groups, the reduction in expenditure contributes to an improvement on pension finances. However, we can observe that in the long run, based on the assumptions of this illustration, even without any pandemic the sustainability of the PAYG Spanish pension scheme is compromised. This is due to forecasts of population pyramids that take into account both ageing and fertility rates, indicating potential challenges in maintaining the scheme’s financial sustainability over time.

## Sensitivity analysis

In previous sections, mortality jumps have been introduced, based on historical data, to occur every 20 years and with a jump-size similar to the COVID-19 pandemic. However, in reality, mortality jump events occur randomly with random jump sizes. Fertility rates can also change during catastrophic events. Therefore, it is necessary to analyse the outcomes across various scenarios: different choices of fertility rates, mortality jump sizes and their frequency, and different levels of unemployment rates.

**Fig. 16:**
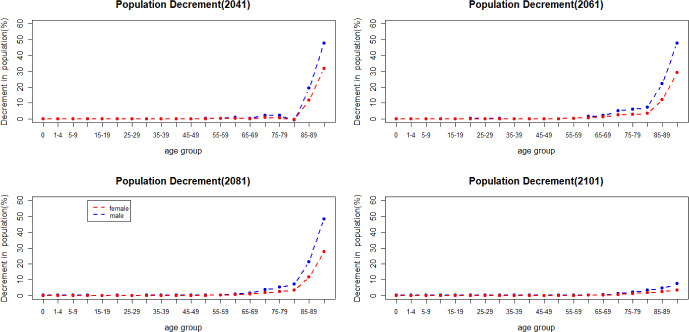
Percentage of population decrement with a mortality jump size of 200$\%$. Mortality rate is capped at 0.99 when analysing larger jump sizes

**Fig. 17:**
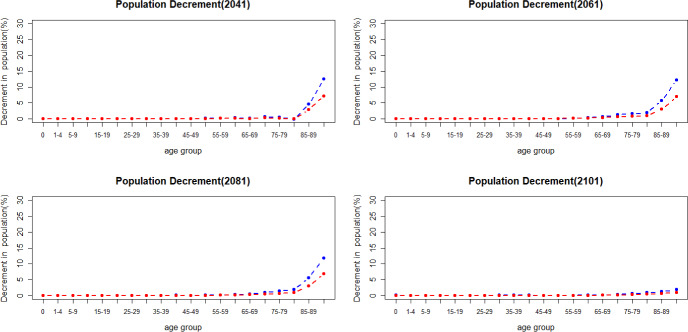
Percentage of population decrement with a jump size of 50$\%$

**Fig. 18:**
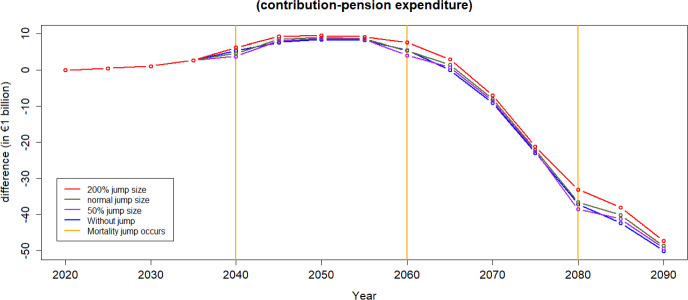
Difference between income from contributions and pension expenditure with different jump sizes

### Different mortality jump sizes

Mortality jump sizes depend on the cause of a catastrophic event and its extent. In this section, we explore results if mortality jumps were doubled or halved compared to our reference scenario. As shown in Figs. 16 and 17, population loss has a similar pattern under different jump sizes, with young age groups remaining relatively unaffected by the variations in jump sizes. The pension financial sustainability, Fig. 18, is affected by the size of the jumps, i.e., larger jumps result in a more substantial reduction in pension expenditure—main driver of the pandemic. Consequently, this leads to an improvement in pension liquidity during the mortality jumps.

#### Different fertility levels

**Fig. 19:**
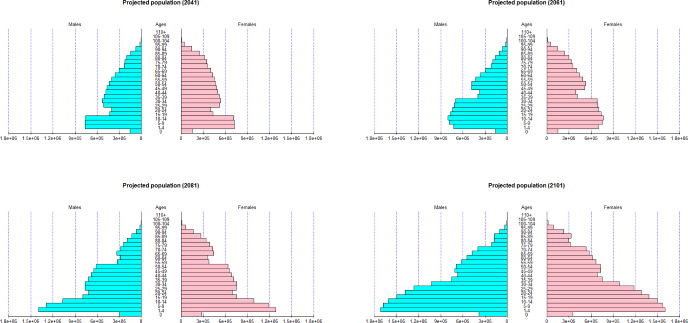
Projected population with 200$\%$ fertility rate

**Fig. 20:**
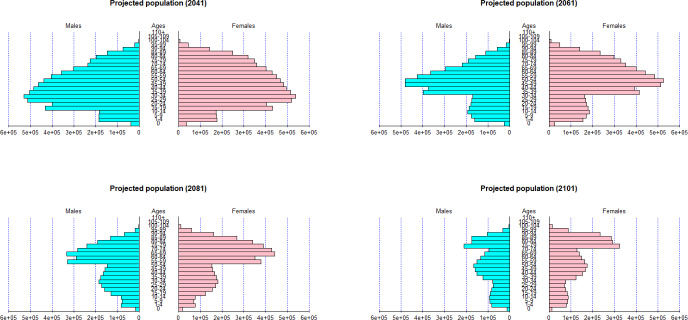
Projected population with 50$\%$ fertility rate

The fertility level may change in the future due to various reasons. Figures 19 and 20 show forecasts of population pyramids based on extremely high and low fertility levels—compared to the baseline scenario—maintaining the same mortality level as in Sect. 3.

Under high levels of fertility, the population size grows, with a predominant portion of the population being younger. Conversely, lower fertility rates leading to an aging society, as evidenced by the values in the old-age dependency ratio values (Tables 3 and 4).

**Table 3:** Old-age dependency ratio with 200$\%$ fertility rate

Gender	2041	2061	2081	2101
Total	0.2505	0.2139	0.2241	0.1963
Female	0.2978	0.2608	0.2733	0.2346
Male	0.1928	0.1549	0.1645	0.1555

**Table 4:** Old-age dependency ratio with 50$\%$ fertility rate

Gender	2041	2061	2081	2101
Total	0.2505	0.3208	0.6585	0.6024
Female	0.2978	0.3844	0.7879	0.7522
Male	0.1928	0.2370	0.4938	0.4358

**Fig. 21:**
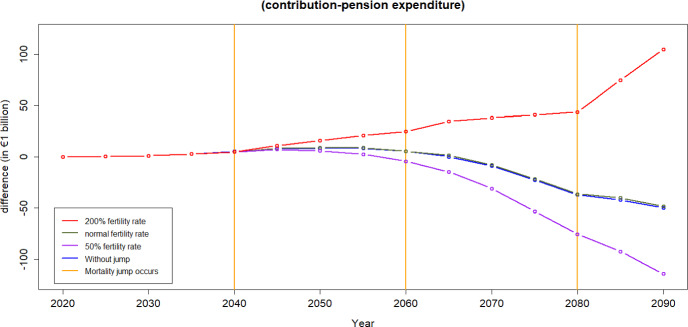
Difference between contribution and expenditure for different fertility rates

As for the population decline caused by mortality jumps, when different fertility levels are used, the decrease in population size during mortality jumps hardly changes for any age group. This is because the size of the mortality jump is relatively small compared to the change in fertility. In addition, the jump in mortality mainly (generally) affects the older groups over 65 years of age, who have fertility rates close to 0$\%$. Long-term sustainability of the pension system is restored when fertility rates are doubled, as depicted in Fig. 21.

#### Fewer mortality jumps

**Fig. 22:**
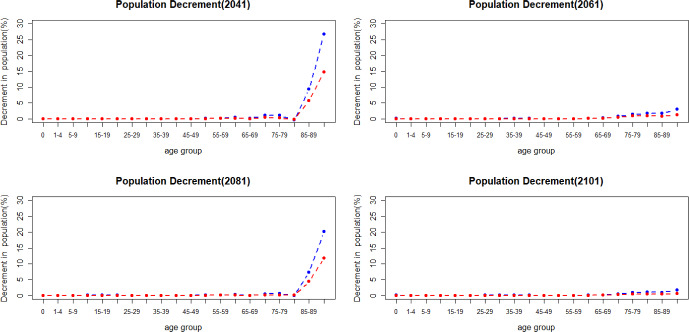
Percentage of population decline with two jumps

**Fig. 23:**
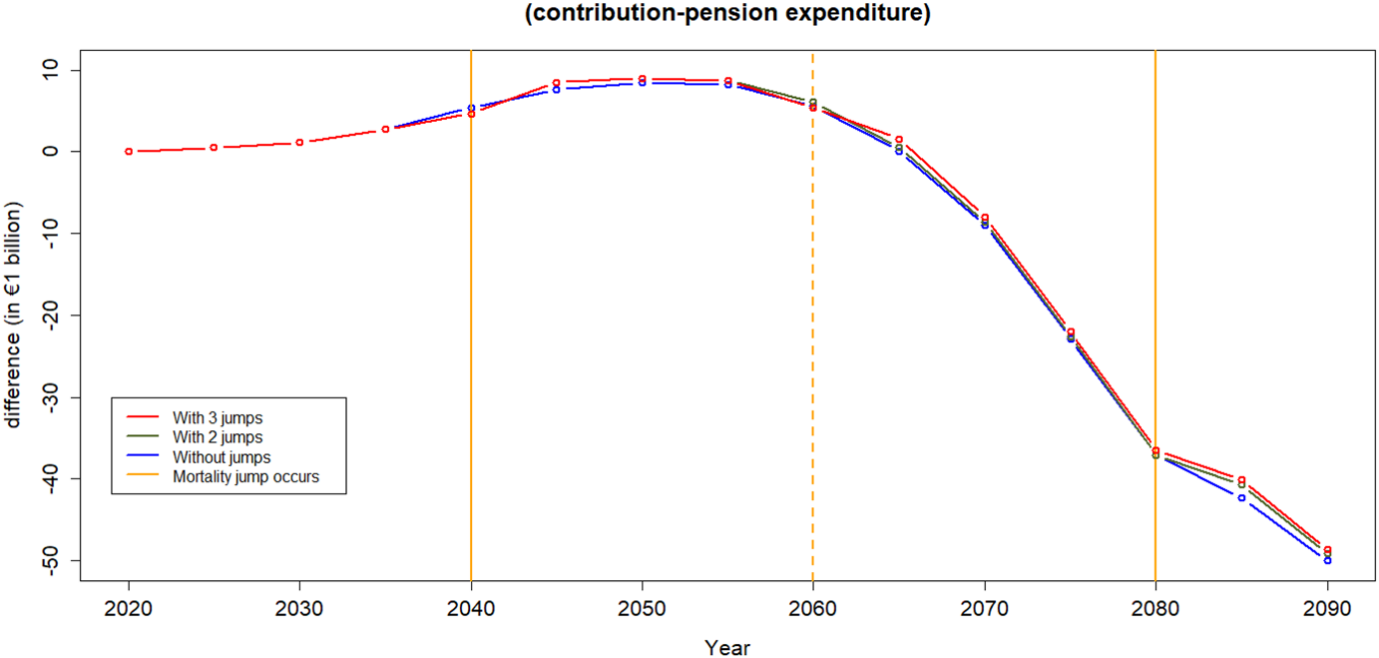
Difference between contribution and expenditure for different numbers of jumps

Figure 22 shows the level of population decline with two mortality jumps, occurring after 20 and 60 years. Figure 23 indicates that with only two anticipated jumps in the future, there will be a diminished increase in the pension scheme’s capital following the second jump. As expected, the population decline levels are negligible in the year 2061 and 2101 (20 years after the jump).

**Fig. 24:**
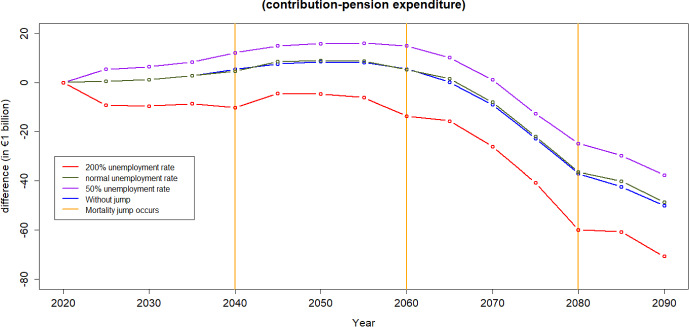
Difference between contribution and expenditure for different levels of unemployment rates

#### Different unemployment levels

Unemployment rates can vary in the future. Figure 24 shows the difference of contribution and expenditure under different levels of unemployment rate *throughout the analysed period*. It is not surprising that the pension scheme’s balance changes drastically under different unemployment levels.

If the unemployment rate doubled that of the baseline scenario *only* for the duration of the pandemic, the income from contributions would decrease, resulting in a new balance of the scheme being 1.19% (1.45%) of the 2022 GDP lower in 2040 (2060) compared to the baseline scenario ( with similar trend to COVID-19). This would jeopardise the financial equilibrium of the scheme during those years immediately after the pandemic. The situation would be even worse if the unemployment rate tripled, with the decrease in contributions (and consequently balance of the scheme) amounting to 1.93% (2.58%) of the 2022 GDP in 2040 (2060)—compared to the baseline scenario.

## Conclusion

Population in most of the Europe is ageing, leading to an increase in dependency ratios—and consequently deteriorating the financial sustainability of pension systems. The COVID-19 pandemic has brought unexpected increases in mortality rates during the past few years—which may also have an impact on population pyramids.

In this study, mortality rates, fertility rates, and population pyramids are projected using Spanish data through the application of functional data analysis. The results of the projected population are in line with the trend of the projected data from Eurostat (2023), i.e. European countries like Spain will face an ageing population and a higher level of old-age dependency ratio in the future.

The incorporation of mortality jumps, sharing characteristics with the COVID-19 pandemic, reveals that the short-term mortality jumps have a greater impact on mortality at older ages, particularly beyond the age of 80. Furthermore, men in the older age groups are more affected than women during catastrophic events—displaying higher mortality rates.

We also explore the financial sustainability of the Spanish public pension scheme considering population projections and mortality jumps caused by future pandemics. In times of pandemics we have two effects that go in different directions in terms of sustainability, these are the increase in mortality—which causes a decrease in pension spending—and the increase in unemployment—which causes a decrease in income from contributions. For the first time to the best of our knowledge, we analyse the consequences of both effects on pension sustainability by employing plausible assumptions and features resembling those of the COVID-19 pandemic. Our findings indicate that, in the event of a pandemic, sustainability is predominantly driven by the mortality effect that generates a decrease in the pension expenditure. In our analysis, we also show that the Spanish PAYG pension scheme is not financially sustainable in the next 80 years considering our population forecasts.

The COVID-19 pandemic caused significant economic and financial disruption. According to Yeyati and Filippini (2021), the pandemic’s shock caused more than 90 percent of the global economy to experience a contraction in per capita GDP, marking the highest share of countries simultaneously contracting since the Great Depression of 1930–1932. Cavallo (2021) notes that the pandemic’s impact on the supply–demand chain and lockdowns led to substantial inflation. Cavallo (2023) further highlights that changes in consumption patterns during the pandemic, such as lockdowns, caused significant inflation in food categories—compared to transportation and other sectors—raising the cost of living and impacting low-income families. Additionally, the pandemic affected financial markets, prompting many countries to implement monetary policies. Wei and Han (2021) report that interest rates were adjusted to near-zero or even negative levels in countries like Australia, Norway and Switzerland to mitigate financial market panic. Changes in economic factors such as inflation and interest rates can affect the financial equilibrium of the pension scheme, potentially increasing pressure to raise pensions to maintain retirees’ purchasing power. However, analysing this from a general equilibrium perspective is beyond the scope of this paper, but exploring these dependencies within an economic model could provide invaluable insights and contribute to the public debate.

From a methodological point of view, it would also be interesting, as a future research, to extend our methodology to incorporate mortality jumps as a stochastic process, such as compound Poisson process with random occurrence time and jump sizes—instead of deterministic values.

## Footnotes

1 Other major reforms include the so-called notional defined contribution pension schemes first developed about two decades age in countries such as Italy, Latvia, Poland and Sweden—see, among others, Holzmann and Palmer (2006), Auerbach and Lee (2009) and Boado-Penas and Vidal-Meliá (2012)—and the complete or partial replacement of PAYG schemes with programs containing a fully funded component as the Chilean case—see, for example, Rofman et al. (2015).

2 Mortality modelling, particularly considering the impact of COVID-19 is challenging and beyond the scope of this paper. Barigou et al. (2020) note that stochastic mortality models, like those by Cairns et al. (2009) and Haberman and Renshaw (2011), focus on fitting historical data rather than predicting future mortality. Barigou et al. (2020) employ regularization techniques to smooth and forecast mortality rates, using a parsimonious decomposition of the mortality surface on a polynomial basis to capture the key features driving mortality patterns. By perturbing mortality data as if there were a pandemic in 2013, they show that their death forecasts are more robust compared to models with a cohort effect. Additionally, the regularized model outperforms the P-spline model in terms of prediction accuracy and stability.

3 This value remains relatively constant, showing consistency whether we examine data from 1960 or 2000.

4 In groups comprising very old ages, mortality rates may exhibit variations among different ages within the group. However, due to the relatively small size of these groups’ population, applying this method is still feasible with acceptable margin of error.

5 www.ine.es.

6 This assumption may appear simplistic. However, we have recalculated our results using the 2022 data on pension value distribution by age group in Spain, and our findings remains robust, with only very minor variations in the deficit of the scheme as a percentage of GDP.

7 Implementing pension growth below the 2% inflation rate would be an unpopular measure for the government. If salaries grow at a higher rate than pension indexation while maintaining the same average pension values and indexation over time, the pension scheme would eventually accumulate a surplus. For example, if salaries grow at an annual rate of 3% (4%) while pension indexation remains at 2%, the balance of the scheme (difference between income and pension spending) in 2060 would be 2.78% (7.73%) of the 2022 GDP.

8 The difference between the income from contributions and pension spending at any given time corresponds to a liquidity indicator.This is a short-term indicator and does not reflect the long-term financial sustainability of the system. In this case, since we calculate the liquidity of the system every year and this is always negative in the future, we can also state that the scheme is not financially sustainable in the long-run.

9 Therefore, this illustration does not aim to assess the financial equilibrium specifically for Spain. Instead, it seeks to examine the effects of a pandemic with similar features to COVID-19 given some plausible population projections for Spain.
